# The Advanced Lung Cancer Inflammation Index Combined With Serum Chloride Levels Predicts the Risk of All-Cause Mortality in Patients With Acute Decompensated Heart Failure (ADHF): A Retrospective Study

**DOI:** 10.31083/RCM39500

**Published:** 2025-10-20

**Authors:** Wenyi Gu, Fazhi Yang, Zijie Liu, Dan Xu, Yun Song, Anyu Xu, Xinuo Ma, Yujuan Peng, Lixing Chen

**Affiliations:** ^1^Department of Cardiology, the First Affiliated Hospital of Kunming Medical University, 650032 Kunming, Yunnan, China; ^2^Yunnan Key Laboratory of Laboratory Medicine, Department of Laboratory Medicine, the First Affiliated Hospital of Kunming Medical University, 650051 Kunming, Yunnan, China; ^3^Centre of Clinical Research and Education, Curtin School of Population Health, Faculty of Health Sciences, Curtin University, 6102 Perth, Australia

**Keywords:** heart failure, inflammation, dystrophy, ALI, electrolytes, prognostic

## Abstract

**Background:**

Serum chloride levels and the advanced lung cancer inflammation index (ALI) score are independent prognostic factors in patients with heart failure (HF). Nevertheless, the interactive relationship between serum chloride levels and the ALI score in predicting all-cause mortality among individuals with acute decompensated heart failure (ADHF) remains undefined.

**Methods:**

The study recruited 1221 patients with ADHF who were hospitalized at the University Affiliated Hospital in China between January 2017 and October 2021. The ALI score was calculated as body mass index × serum albumin level/neutrophil–lymphocyte ratio (NLR), which was used to assess inflammation and nutritional status in ADHF patients.

**Results:**

Following adjustment for confounders including age, sex, New York Heart Association (NYHA) functional classification, left ventricular ejection fraction (LVEF), log-transformed brain natriuretic peptide (lgBNP), and C-reactive protein (CRP) levels, the independent association of ALI score (hazard ratio (HR): 0.984, 95% confidence interval (CI): 0.977–0.990; *p* < 0.0001) and serum chloride (HR: 0.915, 95% CI: 0.897–0.933; *p* < 0.0001) with all-cause mortality persisted. Stratified analysis by ALI score and serum chloride subgroups revealed significant differences in cumulative survival, where lower ALI scores and serum chloride concentrations were associated with a higher risk of all-cause mortality (*p* < 0.0001).

**Conclusions:**

Combining the ALI score with serial serum chloride monitoring adds significant value in predicting all-cause mortality in ADHF patients who may benefit from aggressive chloride correction and anti-inflammatory therapies, potentially modifying the disease trajectory.

## 1. Introduction

Heart failure (HF) is defined as a clinical syndrome with symptoms and/or signs caused by a structural and/or functional cardiac abnormality that is confirmed by elevated natriuretic peptide levels and/or objective evidence of pulmonary or systemic congestion [1]. It is estimated that 64.3 million people worldwide suffered from HF in 2017 [2]. A survey based on medical insurance data from 0.5 billion Chinese urban workers found that the nationally standardized prevalence of HF among people aged 25 years and older in China was 1.1%, with an incidence rate of 275/100,000 person-years. Moreover, the survey estimated that there were 12.05 million existing HF patients and 2.97 million new cases of HF each year. In China, HF patients face alarmingly high rates of mortality and hospital readmission, creating a substantial public health burden that strains healthcare resources and impacts both the quality of life of patients and the national healthcare system. The growing prevalence of HF, particularly among the aging population, has made this condition a critical challenge for healthcare policymakers in China, requiring urgent interventions to improve disease management, optimize treatment protocols, and enhance post-discharge care coordination to reduce the socioeconomic impact on families and the healthcare system [3]. The European Society of Cardiology (ESC) classifies HF into three distinct subtypes according to left ventricular ejection fraction (LVEF), namely, HF with reduced ejection fraction (HFrEF, LVEF ≤40%), mildly reduced ejection fraction (HFmrEF, LVEF 41–49%), and preserved ejection fraction (HFpEF, LVEF ≥50%) [1].

Electrolyte derangements are frequently present in patients with HF. Meanwhile, serum chloride has been recognized as a critical anion with significant implications for understanding the pathophysiological mechanisms of HF. Research indicates that serum chloride plays a pivotal role in maintaining the acid–base balance, modulating neurohormonal activation, and influencing diuretic resistance [4, 5, 6, 7]. Notably, studies have demonstrated that low serum chloride levels are independently associated with adverse clinical outcomes, including increased mortality and hospitalization rates, in HF patients. These findings underscore the potential of serum chloride as both a prognostic biomarker and a therapeutic target in HF management. Although chlorine as a therapeutic target seems reasonable in terms of mechanism, key knowledge gaps remain, and further research is needed to elucidate potential mechanisms and explore chlorine-guided treatment strategies [8, 9, 10, 11, 12, 13, 14].

Malnutrition refers to insufficiencies, surpluses, or imbalances in the intake of energy or nutrients by an individual [15]. Malnutrition is frequently observed in HF patients due to multiple contributing factors, including anorexia, impaired intestinal absorption from gut mucosal edema, systemic inflammation, and the catabolic state induced by HF. These pathological processes collectively drive a progressive nutritional deficit that further exacerbates disease progression and worsens clinical outcomes. This underscores the critical need for comprehensive dietary assessment and targeted interventions in HF management protocols [16]. Serum albumin concentration and body mass index (BMI) values are well-established, clinically accessible markers of nutritional status that have demonstrated strong prognostic value in HF populations. These biomarkers may reflect nutritional depletion and potential disease severity in patients with HF. Meanwhile, these findings emphasize the importance of incorporating routine nutritional assessments into a comprehensive HF risk stratification program [17, 18, 19, 20]. The advanced lung cancer inflammation index (ALI), calculated as BMI × serum albumin/neutrophil-to-lymphocyte ratio (NLR), was originally developed as an integrated biomarker to assess systemic inflammation in oncology patients [21]. Emerging evidence has also demonstrated the significant prognostic utility of this index in HF populations. The ALI may reflect the levels of malnutrition and inflammation in patients with HF. Moreover, the ALI has a significantly higher prognostic stratification ability than single-component indicators, as it synchronously evaluates the multidimensional characteristics of malnutrition and systemic inflammation status. This synergistic effect makes the ALI an ideal tool for identifying high-risk HF patients, providing more reliable risk predictions [22, 23].

Both serum chloride levels and the ALI score have been established as independent prognostic factors in HF patients; however, their potential interaction and combined predictive value for all-cause mortality risk remain poorly understood. Thus, further investigations are needed to determine whether these markers have additive or synergistic effects in risk stratification, which could enhance prognostic accuracy and guide personalized therapeutic strategies for HF management. Further, this study hypothesized a risk interaction between serum chloride levels and the ALI score, proposing that patients with concurrently lower ALI scores and reduced serum chloride levels would exhibit poorer outcomes. Therefore, this study aimed to investigate the predictive value of combining the ALI score and serum chloride levels for all-cause mortality in HF patients.

## 2. Methods

### 2.1 Study Population

**Fig. 1.**
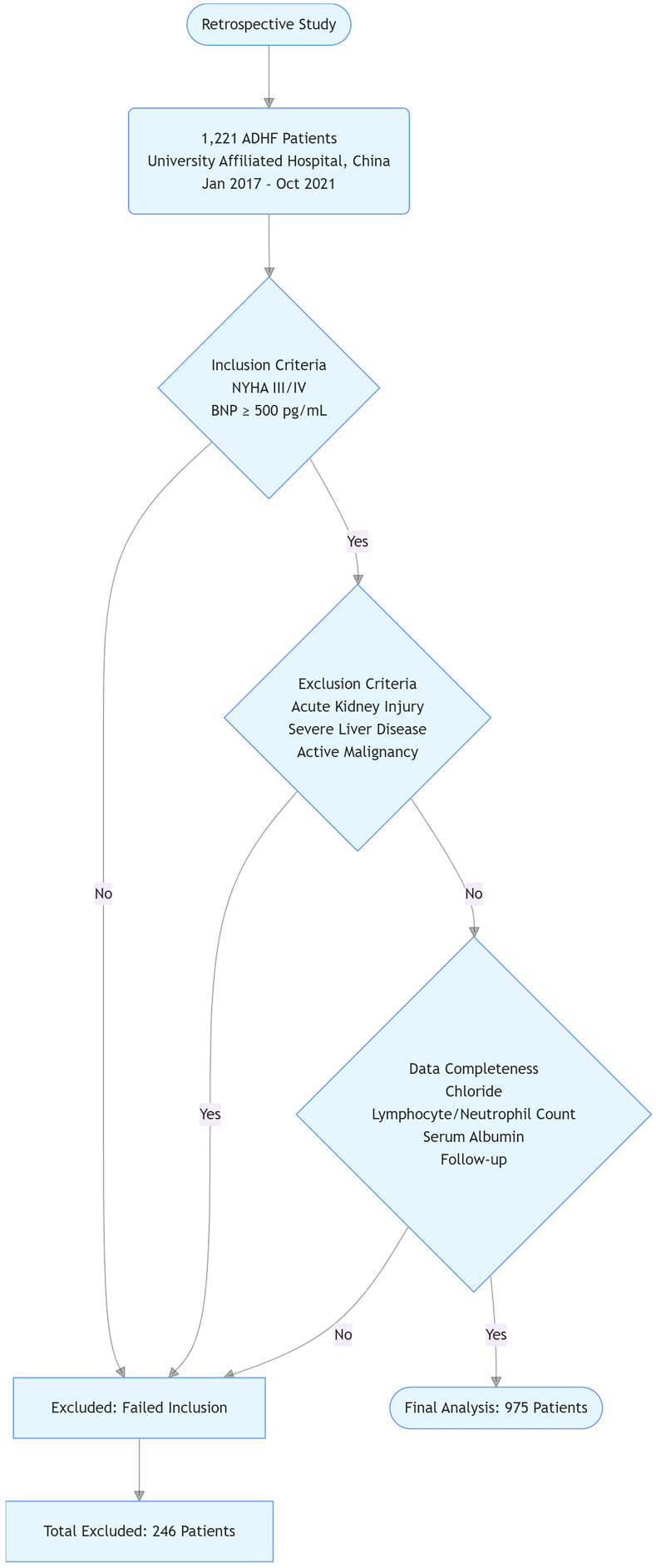
**Study flowchart**. ADHF, acute decompensated heart failure; NYHA, New York Heart Association; BNP, brain natriuretic peptide.

This study retrospectively included 1221 acute decompensated heart failure (ADHF) patients admitted to a university-affiliated hospital in China from January 2017 to October 2021. All patients met the following criteria: (1) diagnosed with New York Heart Association (NYHA) classes III–IV for severe HF; (2) brain natriuretic peptide (BNP) levels ≥500 pg/mL upon admission. Patients were excluded if they had acute kidney injury, severe liver disease, or active malignancy, or if they lacked data on chloride, lymphocyte count, neutrophil count, serum albumin, or follow-up information. After applying these criteria, 975 patients with HF were included in the final analysis (Fig. 1).

### 2.2 Data Collection

The characteristic data for the patients included demographic data, clinical information, medications, and complications at the time of admission. On admission, blood samples were collected to determine BNP, myoglobin, creatine kinase-MB (CK-MB), troponin I, and D-dimer levels before any therapeutic measures were recorded. Following a standardized 10–12-hour fasting period, additional blood specimens were collected in strict adherence to institutional protocols and promptly transported to the central laboratory of the University Affiliated Hospital for analysis using validated methodologies. Laboratory assessments included a complete blood count (white blood cell count, lymphocyte count, red blood cell count, hemoglobin), serum electrolytes (chloride, sodium), albumin, creatinine, uric acid, C-reactive protein (CRP), and lipid profile (total cholesterol (TC), triglycerides (TGs)). The estimated glomerular filtration rate (eGFR) was calculated using the modified Modification of Diet in Renal Disease (MDRD) equation, as follows: eGFR (mL/min/1.73 m^2^) = 194 × (serum creatinine)^-1.094^ × (age)^-0.287^ × 0.739 (for females). Electrocardiographic (ECG) data were acquired using a standardized 12-lead ECG machine. Transthoracic echocardiography was performed within 72 hours of hospital admission to assess cardiac structure and function. In the event of hospital readmission secondary to decompensated HF, the date of the first rehospitalization and corresponding clinical data were systematically documented. All-cause mortality was the primary endpoint of this study, with in-hospital mortality data systematically extracted from the electronic medical record system of the First Affiliated Hospital of Kunming Medical University. Outcome data for discharged patients were collected through a standardized follow-up protocol. Patients were routinely followed up by the researchers, primarily through outpatient visits or telephone contact. For patients who could not be contacted, follow-up was censored at their last documented clinical encounter or the date of their previous responsive contact.

### 2.3 Calculation of NLR and ALI

NLR was quantified as the quotient of peripheral blood neutrophil counts (×10^9^/L) over lymphocyte counts (×10^9^/L). The ALI was calculated using the following equation: ALI = [BMI (kg/m^2^) × serum albumin (g/dL)]/NLR.

### 2.4 Statistical Analysis

Normally distributed variables are presented as the mean ± SD, non-normally distributed variables are presented as the median (interquartile range), and categorical variables are expressed as counts and percentages. Normally distributed variables were compared using one-way analysis of variance (ANOVA), while non-normally distributed variables were compared using the Kruskal–Wallis test, and categorical variables were compared using the chi-square test. Skewed distributions of BNP levels were converted to natural logarithms. Spearman correlation coefficients (r) or Pearson correlation coefficients (r) were used to determine correlations between continuous variables. All clinically relevant parameters were initially analyzed using univariate regression models. Variables with *p*-values of 0.05 in the univariate analyses were included in the multivariate Cox models, using the forward: Likelihood Ratio (LR) method. Univariate and multivariable Cox proportional hazard regression models were constructed to study the predictive value of serum chloride levels and the ALI score as continuous and categorical variables. Estimates of risk are presented as hazard ratios (HRs) with their respective 95% confidence intervals (CIs). Covariates included in the final multivariate model were age, NYHA class IV, ALI score, log-transformed brain natriuretic peptide (lgBNP), hemoglobin level, platelet count, serum chloride level, aspartate transaminase (AST) level, and CRP level. Survival curves were constructed using the Kaplan–Meier method, and intergroup differences were statistically evaluated via the log-rank test. Receiver operating characteristic (ROC) curve analysis was performed to assess the prognostic value of the ALI score combined with serum chloride levels for mortality risk in patients with HF. The studies were performed using SPSS ver. 26 (IBM, Armonk, NY, USA), GraphPad Prism 9.5 (GraphPad Software, San Diego, CA, USA), and the statistical software packages in R 4.2.0 (R Foundation for Statistical Computing, Vienna, Austria).

## 3. Results

### 3.1 Baseline Patient Characteristics

The mean age of the study sample was 66.43 ± 12.83 years, and 60.8% (n = 593) of the patients were male. During a median (p25% to p75%) follow-up of 762 (361–1129) days, 478 (49.03%) patients died.

The cohort was divided into three groups using the ALI tertile cutoffs: a low ALI group (Q1, ≤20.67), an intermediate ALI group (Q2, 20.67–35.66), and a high ALI group (Q3, >35.66). There were no significant differences among the ALI tertiles in gender, smoking status, drinking history, history of hypertension, or history of diabetes. Patients in the lower ALI tertile groups had a higher age, a lower BMI, higher neutrophil counts, lower lymphocyte counts, higher CRP levels, lower blood chloride levels, higher BNP levels, and higher creatinine levels. Table 1 summarizes the baseline clinical characteristics and guideline-directed medical therapies for the 3 ALI subgroups.

**Table 1.** **Baseline characteristics of this study population**.

		ALI (n = 975)	ALI subgroups			*p*-value
			Group 1 (n = 325)	Group 2 (n = 325)	Group 3 (n = 325)	
ALI score		30.13 ± 17.65	12.69 ± 5.01	27.45 ± 4.52	50.23 ± 13.18	<0.0001
Clinical demographics						
	Gender (years)	66.43 ± 12.83	70.38 ± 11.63	65.93 ± 11.82	62.96 ± 13.85	<0.0001
	Male, n (%)	593 (60.8)	210 (64.6)	194 (59.7)	189 (58.2)	0.211
	BMI (kg/m^2^)	23.02 ± 3.84	22.17 ± 3.18	22.92 ± 3.95	23.98 ± 4.13	<0.0001
	SBP (mmHg)	122.20 ± 22.82	119.61 ± 22.37	123.73 ± 23.35	123.26 ± 22.57	0.042
	DBP (mmHg)	76.38 ± 15.22	73.87 ± 14.11	76.67 ± 15.94	78.61 ± 15.21	<0.0001
	NYHA class IV, n (%)	351 (36.0)	143 (44.0)	115 (35.4)	93 (28.6)	<0.0001
Medical history						
	Smoking status, n (%)	322 (33.0)	103 (31.7)	100 (30.8)	119 (36.6)	0.234
	Drinking status, n (%)	160 (16.4)	46 (14.2)	52 (16.0)	62 (19.1)	0.231
	DM, n (%)	261 (26.8)	95 (29.2)	81 (24.9)	85 (26.2)	0.442
	Hypertension, n (%)	531 (54.5)	179 (55.1)	178 (54.8)	174 (53.5)	0.917
	CHD, n (%)	478 (49.0)	188 (57.8)	147 (45.2)	143 (44.0)	<0.0001
	AF, n (%)	332 (34.1)	114 (35.1)	104 (32.0)	114 (35.1)	0.633
Grouped according to LVEF						0.385
	HFrEF, n (%)	439 (45.0)	132 (40.6)	155 (47.7)	152 (46.8)	
	HFmrEF, n (%)	171 (17.5)	59 (18.2)	55 (16.9)	57 (17.5)	
	HFpEF, n (%)	365 (37.4)	134 (41.2)	115 (35.4)	116 (35.7)	
Laboratory data						
	WBC (10^9^/L)	6.69 (5.42, 8.52)	8.14 (6.37, 10.95)	6.40 (5.33, 7.71)	6.11 (5.18, 7.38)	<0.0001
	Neutrophils (10^9^/L)	5.15 ± 2.88	7.23 ± 3.78	4.51 ± 1.46	3.71 ± 1.32	<0.0001
	Lymphocytes (10^9^/L)	1.51 ± 0.68	1.06 ± 0.46	1.47 ± 0.43	1.99 ± 0.75	<0.0001
	RBCs (10^12^/L)	4.56 (4.10, 5.04)	4.41 (3.95, 4.88)	4.61 (4.14, 5.06)	4.69 (4.29, 5.16)	<0.0001
	HB (g/L)	140 (125, 154)	137 (120, 151)	141 (127, 154)	144 (130, 156)	<0.0001
	PLTs (10^9^/L)	200.14 ± 79.26	200.61 ± 87.09	202.86 ± 75.50	196.96 ± 74.71	0.632
	Albumin (g/dL)	3.67 (3.42, 3.99)	3.53 (3.25, 3.80)	3.68 (3.43, 3.96)	3.84 (3.55, 4.16)	<0.0001
	CRP (mg/L)	7.20 (3.00, 20.50)	14.70 (5.37, 33.61)	6.75 (2.81, 17.57)	4.50 (2.20, 12.25)	<0.0001
	Fib (g/L)	3.51 ± 1.20	3.81 ± 1.44	3.50 ± 1.10	3.21 ± 0.95	<0.0001
	lgBNP	3.17 ± 0.28	3.22 ± 0.30	3.16 ± 0.25	3.12 ± 0.28	<0.0001
	Potassium (mmol/L)	3.93 ± 0.59	3.94 ± 0.66	3.91 ± 0.57	3.92 ± 0.53	<0.854
	Sodium (mmol/L)	141.01 ± 4.45	139.79 ± 4.90	141.26 ± 4.19	141.96 ± 3.93	<0.0001
	Chlorine (mmol/L)	102.97 ± 4.63	101.50 ± 5.13	103.44 ± 4.43	103.96 ± 3.89	<0.0001
	ALT (IU/L)	24.90 (16.50, 41.60)	24.60 (16.20, 42.15)	22.90 (15.45, 40.10)	25.40 (17.90, 43.05)	0.223
	AST (IU/L)	28.20 (20.00, 42.20)	30.20 (20.95, 52.70)	27.00 (20.00, 40.10)	27.60 (20.25, 39.30)	0.019
	Cre, (µmol/L)	102.50 (83.00, 132.00)	109.20 (85.80, 152.50)	104.60 (83.05, 129.85)	94.60 (79.70, 117.40)	<0.0001
	SUA (µmol/L)	481.00 (380.20, 594.65)	480.85 (380.58, 602.48)	497.90 (380.00, 596.60)	475.70 (378.78, 586.20)	0.963
	GFR (mL/min)	44.56 (32.73, 57.22)	38.80 (27.11, 51.44)	43.16 (34.15, 56.39)	38.40 (51.41, 62.81)	<0.0001
	FBG (mmol/L)	5.96 ± 3.15	6.23 ± 3.39	5.95 ± 3.02	5.69 ± 3.02	0.092
	TC (mmol/L)	3.56 (2.94, 4.22)	3.41 (2.80, 4.13)	3.65 (3.02, 4.26)	3.62 (3.08, 4.29)	0.002
	TG (mmol/L)	1.10 (0.86, 1.52)	1.06 (0.85, 1.40)	1.07 (0.84, 1.45)	1.20 (0.88, 1.62)	0.004
	HDL-C (mmol/L)	0.96 (0.79, 1.17)	0.94 (0.76, 1.15)	0.96 (0.80, 1.15)	0.99 (0.82, 1.19)	0.064
	LDL-C (mmol/L)	2.18 (1.66, 2.79)	2.13 (1.52, 2.79)	2.22 (1.73, 2.83)	2.21 (1.69, 2.76)	0.131
Echocardiography						
	HR (beat/minute)	84.56 ± 20.80	86.53 ± 21.85	81.66 ± 18.45	85.48 ± 21.68	0.007
	LVEF (%)	42 (32, 57)	45 (33, 58)	41 (31, 58)	42 (32, 56)	0.096
Treatment						
	CRT, n (%)	101 (10.4)	44 (13.5)	27 (8.3)	30 (9.2)	0.065
	Dapagliflozin, n (%)	211 (21.6)	66 (20.3)	71 (21.8)	74 (22.8)	0.744
	Beta blockers, n (%)	644 (66.1)	200 (61.5)	216 (66.5)	228 (70.2)	0.067
	ACEI/ARB/ARNI, n (%)	530 (54.4)	169 (52.0)	184 (56.6)	177 (54.5)	0.497
	Diuretics, n (%)	850 (87.2)	290 (89.2)	273 (84.0)	287 (88.3)	0.104
	Spironolactone, n (%)	815 (83.6)	278 (85.5)	268 (82.5)	269 (82.8)	0.506

Abbreviations: ALI, advanced lung cancer inflammation index; BMI, body mass index; SBP, systolic blood pressure; DBP, diastolic blood pressure; NYHA class, New York Heart Association cardiac function classification; CHD, coronary heart disease; AF, atrial fibrillation; CRT, cardiac resynchronization therapy; WBC, white blood cell; HB, hemoglobin; PLT, platelet; Cre, Creatinine; SUA, serum uric acid; GFR, glomerular filtration rate; FBG, fasting blood glucose; CRP, C reactive protein; lgBNP, log-transformed brain natriuretic peptide; LVEF, left ventricular ejection fraction; ACEI, angiotensin-converting enzyme inhibitor; ARB, angiotensin II receptor blocker; ARNI, angiotensin receptor neprilysin inhibitor; DM, diabetes mellitus; HFrEF, heart failure with reduced ejection fraction; HFmrEF, heart failure with mildly reduced ejection fraction; HFpEF, heart failure with preserved ejection fraction; RBC, red blood cell; Fib, fibrinogen; ALT, alanine aminotransferase; AST, aspartate transaminase; TC, total cholesterol; TG, triglyceride; HDL-C, high density lipoprotein cholesterol; LDL-C, low density lipoprotein cholesterol; HR, hazard ratio.

### 3.2 Prognostic Value of the ALI Score for All-Cause Mortality in HF Patients 

**Fig. 2.**
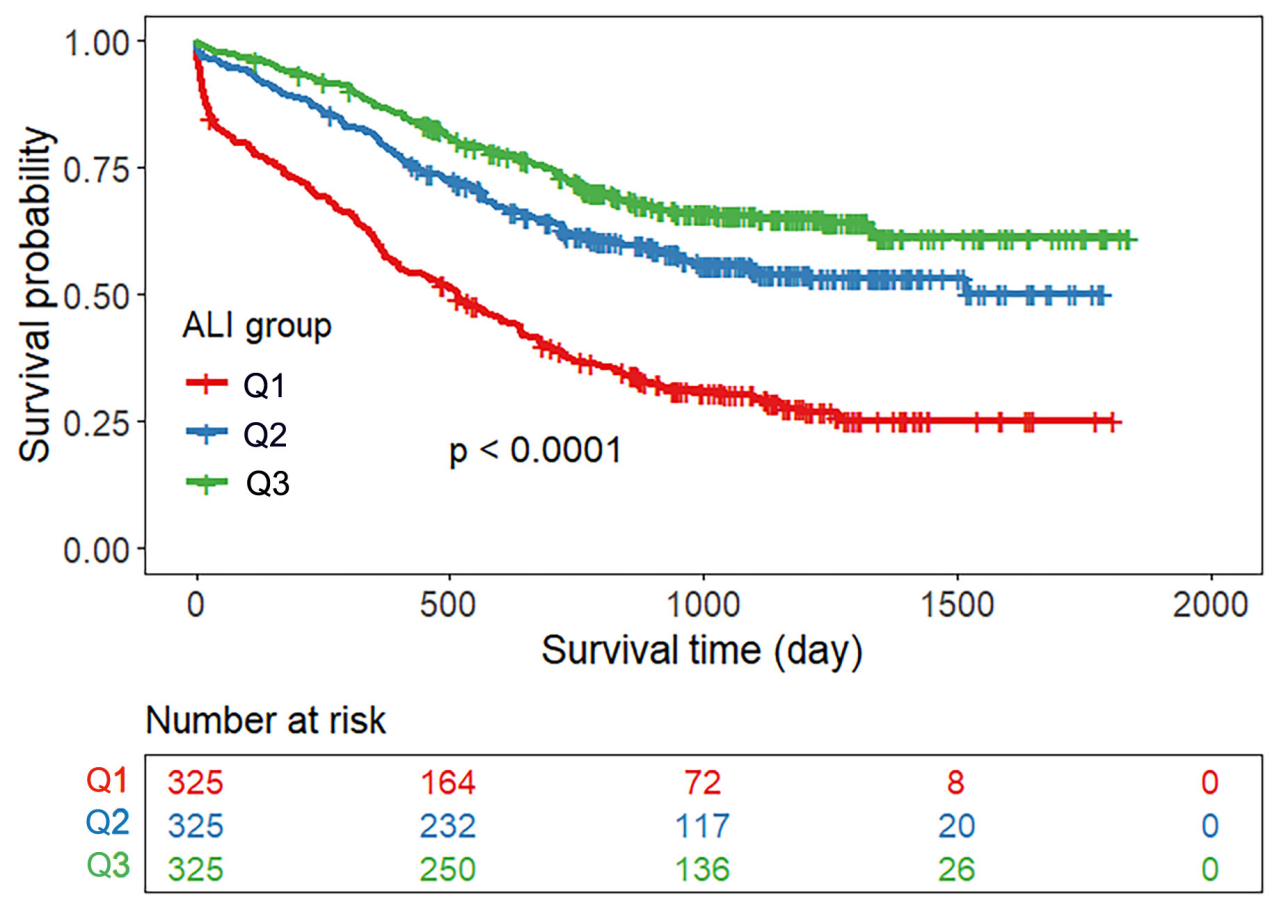
**Kaplan‒Meier survival curve for all-cause mortality according to the ALI**. Note: Q1: ALI ≤20.67; Q2: 20.67 > ALI ≤ 35.66, Q3: ALI >35.66.

**Fig. 3.**
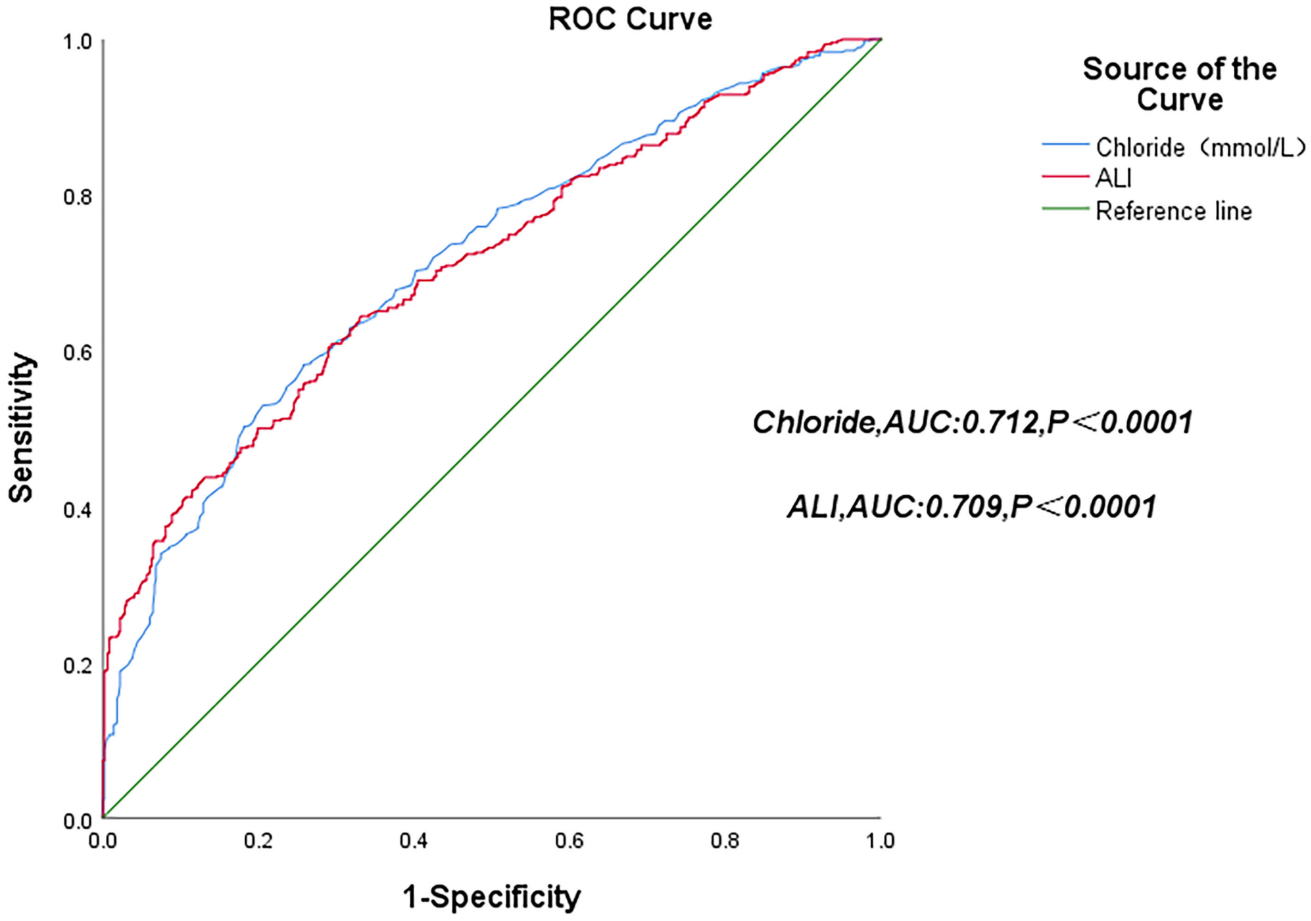
**ROC curves of serum chloride levels and the ALI score**. ROC, receiver operating characteristic; AUC, area under the curve.

Patients were divided into three groups according to the tertiles of the ALI score. Fig. 2 shows that the cumulative survival rate of patients was lowest in the lowest ALI quartile group, and the mortality risk decreased gradually among the three groups (log-rank χ^2^ = 116.196; *p* < 0.0001). The prognostic value of the ALI score for all-cause mortality in patients with HF was studied by generating ROC curves (Fig. 3). The area under the curve (AUC) was 0.709 (95% CI: 0.676–0.741, *p* < 0.0001).

The univariate and multivariate Cox proportional hazards models indicated a negative correlation between the admission ALI score and the risk of all-cause mortality (Table 2). An increase in the ALI score was associated with a 3.8% lower risk of all-cause mortality (95% CI: 0.955–0.968; *p* < 0.0001). After multivariate adjustment, this correlation remained stable (95% CI: 0.973–0.986; *p* < 0.0001) (Table 3).

**Table 2.** **Univariable and multivariable Cox proportional hazards predictive model for all-cause mortality in patients with HF**.

	Univariable		Multivariable	
	HR (95% CI)	*p-*value	HR (95% CI)	*p*-value
Age	1.034 (1.026, 1.042)	<0.0001	1.024 (1.016, 1.033)	<0.0001
Male vs. female	0.986 (0.820, 1.185)	0.880		
Coronary heart disease	0.940 (0.785, 1.124)	0.497		
Hypertension	0.958 (0.800, 1.147)	0.641		
Diabetes mellitus	0.793 (0.653, 0.964)	0.020		
AF	0.879 (0.729, 1.059)	0.176		
Smoking status	0.986 (0.816, 1.193)	0.888		
Drinking status	1.222 (0.950, 1.572)	0.112		
NYHA class IV	2.286 (1.910, 2.736)	<0.0001	1.506 (1.246, 1.820)	<0.0001
ALI score	0.962 (0.955, 0.968)	<0.0001	0.984 (0.977, 0.990)	<0.0001
LVEF	0.995 (0.989, 1.000)	0.056		
lgBNP	5.771 (4.115, 8.095)	<0.0001	3.490 (2.468, 4.936)	<0.0001
WBCs	1.079 (1.054, 1.104)	<0.0001		
RBCs	0.736 (0.651, 0.832)	<0.0001		
HB	0.990 (0.986, 0.994)	<0.0001	0.992 (0.988, 0.996)	<0.0001
PLTs	0.998 (0.997, 0.999)	0.002	0.999 (0.997, 1.000)	0.012
Fib	1.093 (1.015, 1.177)	0.019		
Potassium	1.175 (1.006, 1.373)	0.042		
Chloride	0.878 (0.861, 0.895)	<0.0001	0.915 (0.897, 0.933)	<0.0001
Sodium	0.905 (0.887, 0.923)	<0.0001		
ALT	1.003 (1.002, 1.004)	<0.0001		
AST	1.005 (1.004, 1.006)	<0.0001	1.005 (1.003, 1.010)	0.036
Cre	1.002 (1.002, 1.003)	<0.0001		
SUA	1.001 (1.001, 1.002)	<0.0001		
GFR	0.976 (0.971, 0.981)	<0.0001		
FBG	1.054 (1.029, 1.080)	<0.0001		
TC	0.810 (0.737, 0.891)	<0.0001		
TGs	0.825 (0.707, 0.963)	0.015		
HDL-C	0.628 (0.459, 0.858)	0.004		
LDL-C	0.809 (0.723, 0.904)	<0.0001		
CRP	1.013 (1.011, 1.015)	<0.0001	1.008 (1.005, 1.010)	<0.0001

Note: The univariate Cox proportional hazards model was used to screen variables, and then a multivariate Cox proportional hazards model was constructed. HF, heart failure; HR, hazard ratio; CI, confidence interval.

**Table 3.** **Associations between the ALI score, serum chloride levels, and all-cause mortality**.

Variable		Unadjusted		Model 1		Model 2		Model 3	
		HR (95% CI)	*p*-value	HR (95% CI)	*p*-value	HR (95% CI)	*p*-value	HR (95% CI)	*p*-value
Continuous variables									
	ALI score	0.962 (0.955, 0.968)	<0.0001	0.966 (0.959, 0.973)	<0.0001	0.961 (0.955, 0.968)	<0.0001	0.979 (0.973, 0.986)	<0.0001
	Chloride level	0.878 (0.861, 0.895)	<0.0001	0.882 (0.866, 0.899)	<0.0001	0.877 (0.861, 0.894)	<0.0001	0.909 (0.891, 0.927)	<0.0001
Tripartite variable									
	Chloride level (Q2)	Reference		Reference		Reference		Reference	
	Chloride level (Q1)	2.467 (2.043, 2.980)	<0.0001	2.533 (2.097, 3.060)	<0.0001	2.469 (2.044, 2.982)	<0.0001	1.994 (1.643, 2.421)	<0.0001
	ALI score (Q1)	Reference		Reference		Reference		Reference	
	Q2	0.465 (0.377, 0.573)	<0.0001	0.517 (0.418, 0.640)	<0.0001	0.464 (0.376, 0.573)	<0.0001	0.643 (0.516, 0.800)	<0.0001
	Q3	0.328 (0.260, 0.412)	<0.0001	0.387 (0.306, 0.489)	<0.0001	0.327 (0.260, 0.411)	<0.0001	0.507 (0.398, 0.647)	<0.0001

Adjusted Model 1: adjusted for age; Adjusted Model 2: adjusted for gender; Adjusted Model 3: adjusted for age, gender, coronary heart disease, hypertension, diabetes mellitus, NYHA IV, LVEF, lgBNP, ALI, chloride, and CRP level.

### 3.3 Association Between Serum Chloride Levels and All-Cause Mortality Risk in Patients with HF

**Fig. 4.**
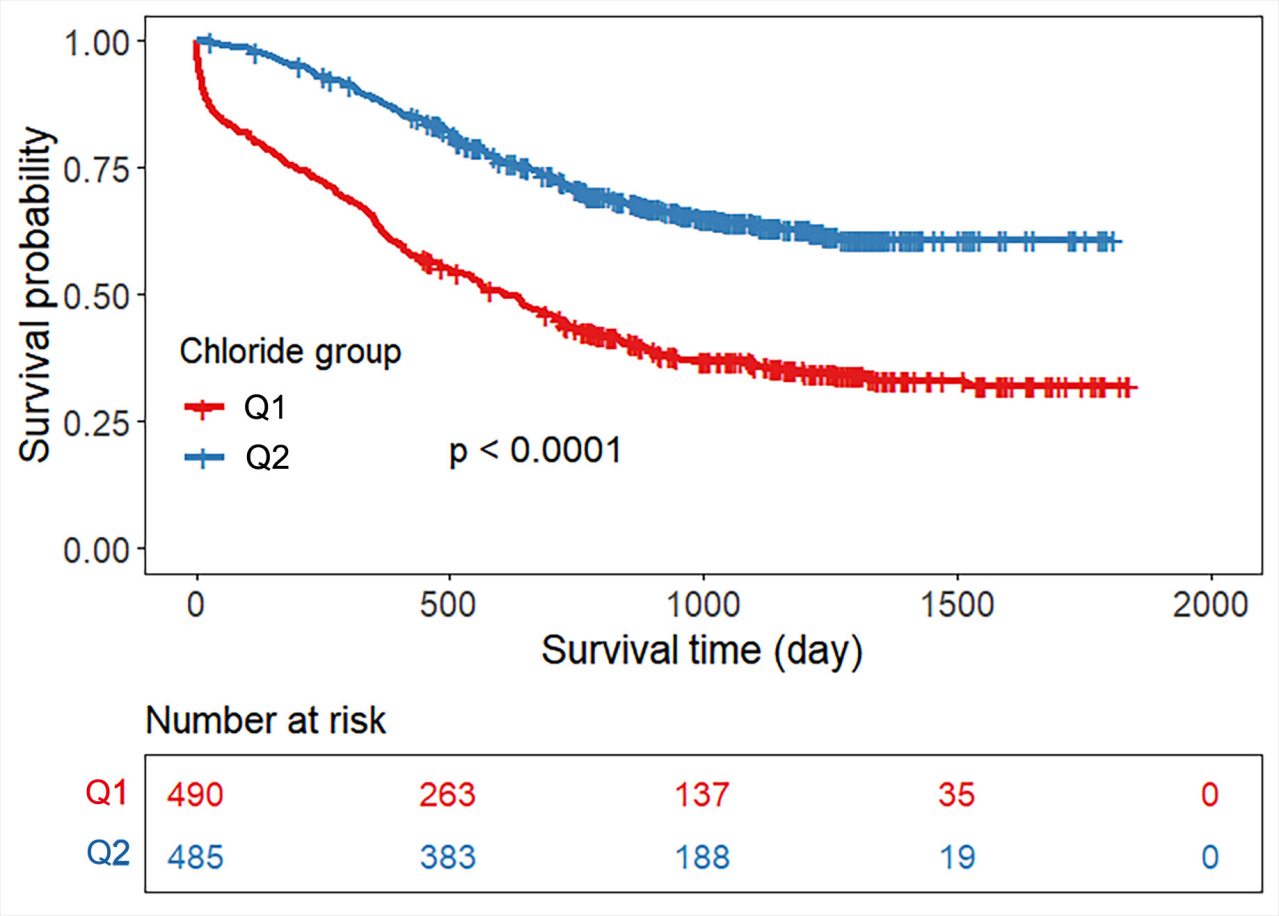
**Kaplan‒Meier survival curve based on serum chloride for all-cause mortality**. Note: Q1: chloride ≤103.3 mmol/L, Q2: chloride >103.3 mmol/L.

Patients were dichotomized at the median serum chloride concentration, resulting in two groups: low-chloride and high-chloride. Kaplan–Meier analysis demonstrated significantly higher all-cause mortality in the hypochloremia group compared to the normochloremia group (log-rank χ^2^ = 93.987; *p* < 0.0001) (Fig. 4). Meanwhile, an ROC curve was generated to study the prognostic value of serum chloride concentrations for all-cause mortality in patients with HF (Fig. 4). The optimal serum chloride cutoff value for predicting all-cause mortality was 102.36 mmol/L. The AUC was 0.712 (95% CI: 0.680–0.744; *p* < 0.0001), with a sensitivity of 58.2% and specificity of 74.2%.

Both univariate and multivariate Cox regression confirmed serum chloride levels as an independent inverse predictor of all-cause mortality (Table 2). An additional 1 mmol/L of chloride was associated with a 12.2% lower risk of all-cause mortality (95% CI: 0.861–0.895; *p* < 0.0001). After multivariate adjustment, this correlation remained stable (95% CI: 0.891–0.927; *p* < 0.0001) (Table 3).

### 3.4 Correlates of the ALI Score

Pearson’s correlation analysis revealed a positive correlation between the ALI score and serum sodium and chloride levels. However, the ALI score was negatively correlated with age, lgBNP, platelet (PLT), Fib, and potassium levels. The details are shown in Table 4.

**Table 4.** **Relationship between the ALI score, serum chloride levels, and baseline characteristics**.

Variable	ALI score		Chloride levels	
	r	*p* value	r	*p* value
Age	–0.264	<0.0001	–0.023	0.465
ALI score	1		0.235	<0.0001
LVEF	–0.078	0.014	0.060	0.061
lgBNP	–0.163	<0.0001	–0.134	<0.0001
WBCs	–0.358	<0.0001	–0.173	<0.0001
RBCs	0.196	<0.0001	–0.071	<0.0001
HB	0.186	<0.0001	–0.070	0.029
PLTs	–0.032	0.318	–0.010	0.754
Fib	–0.226	<0.0001	–0.094	0.003
Potassium	–0.006	0.843	–0.057	0.074
Chloride	0.235	<0.0001	1.000	
Sodium	0.217	<0.0001	0.577	<0.0001
ALT	0.034	0.292	–0.046	0.147
AST	–0.096	0.003	–0.140	<0.0001
Cre	–0.191	<0.0001	–0.081	0.011
SUA	–0.010	0.757	–0.102	0.002
CRP	–0.345	<0.0001	–0.279	<0.0001

Note: The symbol “r” denotes the correlation coefficient. The value ranges of both Pearson correlation coefficient and Spearman correlation coefficient are [–1, 1].

Spearman’s correlation analysis showed that the ALI score was positively correlated with RBC counts, ALT, and HB levels. However, the ALI score was negatively correlated with LVEF, WBC counts, and AST, Cre, SUA, and CRP levels. The details are shown in Table 4.

### 3.5 Correlations of Serum Chloride Levels

Pearson’s correlation analysis showed that serum chloride levels were positively correlated with serum sodium levels and the ALI score. However, serum chloride levels were negatively correlated with age, lgBNP, PLT, Fib levels, and potassium levels. The details are presented in Table 4.

The Spearman correlation analysis revealed a positive correlation between serum chloride and LVEF. However, serum chloride levels were negatively correlated with RBC counts, WBC counts, and HB, ALT, AST, Cre, SUA, and CRP levels. The details are shown in Table 4.

### 3.6 ALI Score Combined with Serum Chloride Levels for Predicting the Risk of All-Cause Mortality in Patients with HF

After stratifying patients with HF by age (interaction *p* = 0.716), gender (interaction *p* = 0.002), serum chloride levels (interaction *p* < 0.0001), and BMI (interaction *p* < 0.0001), a significant interaction was found between the ALI score and serum chloride levels (Table 5).

**Table 5.** **Stratified associations between the ALI score and age, gender, BMI, and chloride levels**.

		ALI score		
		HR (95% CI)	*p*-value	*p* for interaction
Age				0.716
	≤60 yrs	0.995 (0.990, 1.000)	0.044	
	61–70 yrs	0.992 (0.988, 0.996)	<0.0001	
	>70 yrs	0.996 (0.994, 0.998)	<0.0001	
Gender				0.002
	Male	Reference		
	Female	0.921 (0.845, 1.004)		0.061
BMI				<0.0001
	<23 kg/m^2^	0.986 (0.979, 0.994)	<0.0001	
	23–24.9 kg/m^2^	0.977 (0.966, 0.989)	<0.0001	
	≥25 kg/m^2^	0.977 (0.968, 0.986)	<0.0001	
Chloride				<0.0001
	Q1	0.994 (0.992, 0.995)	<0.0001	
	Q2	0.998 (0.996, 1.000)	0.044	

Adjusted for coronary heart disease, hypertension, diabetes mellitus, LVEF, lgBNP, and CRP level. yrs, years.

**Fig. 5.**
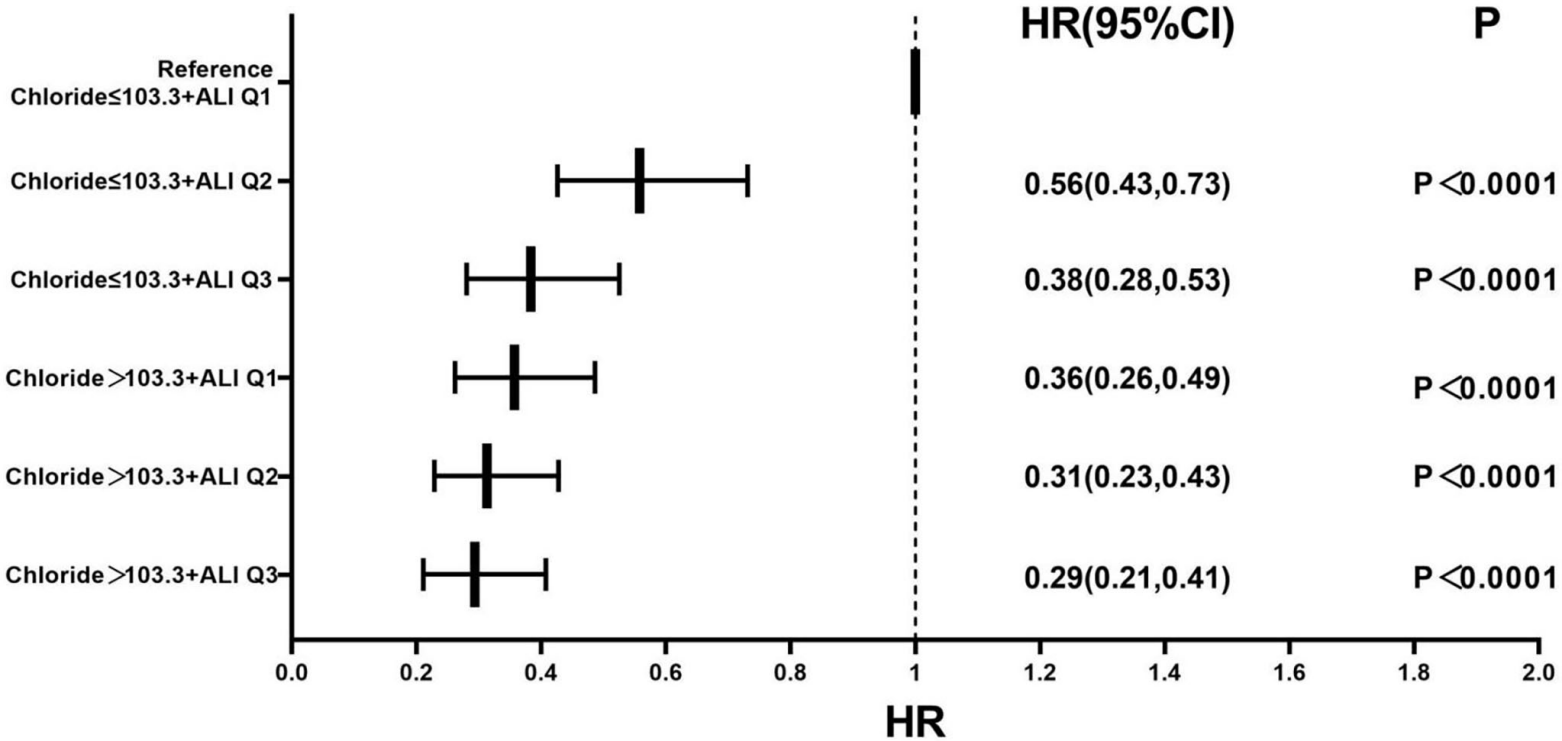
**Forest plot of the multivariable-adjusted all-cause mortality rate in the serum chloride, ALI, and combined groups**. The multivariate Cox regression model was adjusted for age, gender, coronary heart disease, hypertension, diabetes mellitus, NYHA class IV, LVEF, lgBNP, and CRP level. Note: Q1: ALI ≤20.67, Q2: 20.67 > ALI ≤ 35.66, Q3: ALI >35.66.

Based on the tertiles of the ALI and the median serum chloride level, patients were cross-combined into six groups (Table 6). In our Cox proportional hazards analysis, we established Group 1 (chloride level ≤103.3 + ALI Q1 (lowest quartile)) as the reference group to evaluate comparative mortality risks across the stratified patient categories, and HR values were calculated. Subgroup analyses revealed that after adjusting for age, gender, coronary heart disease, hypertension, diabetes mellitus, NYHA class IV, LVEF, lgBNP, and CRP levels, HR values in the first to fifth groups decreased gradually (Fig. 5).

**Table 6.** **Univariable and multivariable Cox proportional hazards analyses of the ALI score combined with serum chloride levels for all-cause mortality**.

	Unadjusted		Adjusted	
	HR (95% CI)	*p*-value	HR (95% CI)	*p*-value
Chloride level ≤ 103.3 + ALI Q1	Reference			
Chloride level ≤ 103.3 + ALI Q2	0.43 (0.33, 0.55)	<0.0001	0.56 (0.43, 0.73)	<0.0001
Chloride level ≤ 103.3 + ALI Q3	0.24 (0.18, 0.33)	<0.0001	0.38 (0.28, 0.53)	<0.0001
Chloride level > 103.3 + ALI Q1	0.29 (0.21, 0.39)	<0.0001	0.36 (0.26, 0.49)	<0.0001
Chloride level > 103.3 + ALI Q2	0.20 (0.15, 0.26)	<0.0001	0.31 (0.23, 0.43)	<0.0001
Chloride level > 103.3 + ALI Q3	0.17 (0.13, 0.26)	<0.0001	0.29 (0.21, 0.41)	<0.0001

Adjusted for age, gender, coronary heart disease, hypertension, diabetes mellitus, NYHA class IV, LVEF, lgBNP, and CRP level.

## 4. Discussion

This study evaluated the association between the admission ALI score and prognostic outcomes in patients with ADHF. We found that the risk of all-cause mortality was higher in patients in the lowest ALI quartile group compared to the higher ALI quartile groups and that the combination of the ALI score and serum chloride levels contributed to risk stratification. To our knowledge, this represents the first investigation to integrate the inflammatory–nutritional spectrum captured by the ALI with pathophysiological pathways for chloride in mortality risk stratification for ADHF. This synergistic approach addresses both metabolic derangements and systemic inflammation simultaneously. Our results demonstrate that this novel biomarker combination provides superior risk stratification compared to either marker alone, addressing critical gaps in existing prognostic models.

The ALI is a simple, reproducible, and cost-effective prognostic tool used to evaluate new prognostic indicators for diseases such as cancer. The ALI integrates three commonly measured clinical parameters: NLR, BMI, and serum albumin, all of which are associated with poor prognosis and may reflect malnutrition and inflammation levels in HF patients. Prior studies have established the independent prognostic value of the ALI in HF, including the demonstration by Maeda *et al*. [23] of its association with mortality and readmission in ADHF and the application by Kurkiewicz *et al*. [24] for risk stratification in advanced HF.

Patients with HF are frequently malnourished due to gastrointestinal oedema, anorexia leading to reduced nutrient intake or inadequate absorption, inflammatory cytokine-induced hypercatabolic syndrome, abnormal liver function due to hepatic congestion, and insulin resistance [25, 26, 27]. The BMI is a predictor of poor prognosis in HF patients, and it is easy to measure BMI clinically; however, it is not an ideal indicator of the nutritional status of patients with HF since these patients usually suffer from water and sodium retention, which leads to short-term changes in body weight; therefore, the BMI is unable to differentiate between weight due to excess fluids and fat. In patients with HF, low BMI is associated with poor prognosis, a phenomenon known as the “obesity paradox” [28, 29]. In our study, patients with HF in the low ALI quartile (Group 1: 22.17 ± 3.18 kg/m^2^) had a lower BMI than those in the other two groups (Group 2: 22.92 ± 3.95 kg/m^2^; Group 3: 23.98 ± 4.13 kg/m^2^; *p* < 0.0001). Hepatic congestion reduces albumin synthesis and lipid transport and synthesis, leading to metabolic disorders [30]. In our study, we found that serum albumin levels were lower in the low ALI quartile group than in the other two groups (*p* < 0.0001). In contrast, the causes of reduced albumin synthesis in the liver include malnutrition, inflammation, and liver dysfunction due to congestion and hypoperfusion [18, 31]. Inflammation also plays a pivotal role in the pathogenesis and progression of HF. NLR, a well-established marker of systemic inflammation, is associated with adverse clinical outcomes in HF patients [32, 33]. The proinflammatory environment in HF accelerates the catabolic process, leading to hypoalbuminemia due to protein breakdown, and induces insulin resistance, anorexia, and impaired nutrient absorption, collectively driving weight loss and cardiac cachexia [34]. Given the multifactorial nature of HF progression, reliance on a single inflammatory marker may provide an incomplete risk assessment. The ALI score is calculated as BMI × serum albumin level/NLR, and a comprehensive evaluation combining these parameters provides an improved prediction of long-term prognosis in patients.

This study demonstrated that the combination of the ALI score and serum chloride levels was valuable for risk stratification in HF patients. Our findings showed that lower chloride levels upon admission were associated with an elevated mortality risk and provided substantial insights into the interpretation of electrolyte disturbance in ADHF. To commence with, our multivariate analyses revealed that serum chloride levels were independently and inversely correlated with mortality, even after adjusting for other prognostic determinants. Secondly, a significant association was observed between serum sodium levels and serum chloride levels (r = 0.577; *p* < 0.0001) (Table 4). Notably, our study found no significant correlation between serum sodium levels and mortality when serum chloride levels were added to the multivariate model (Table 2). These outcomes emphasize the prognostic significance of serum chloride levels in ADHF, suggesting that serum chloride levels provide more robust prognostic evidence than serum sodium levels. These findings also reveal that serum chloride levels may provide important prognostic information for patients with ADHF, highlighting the need for a better understanding of the potential benefits of strategies that can maintain electrolyte homeostasis, particularly long-term diuretic strategies. Low serum chloride levels are a commonly presented electrolyte disturbance in HF patients. The main contributing factors to decreased serum chloride levels are related to the loss of chloride anions in the gastrointestinal tract or kidneys. The chloride absorption in patients with HF may be compromised due to visceral circulation congestion and ensuing intestinal wall edema and barrier dysfunction [35]. In addition, chloride plays a vital role in fluid homeostasis, neurohormonal activation, and diuretic resistance [4], which are commonly acknowledged as key factors in the genesis and progression of HF. Chloride serves as the principal regulator of renin release and tubuloglomerular feedback within the kidneys, while also playing a central role in modulating sodium transport mechanisms in both the thick ascending limb of the loop of Henle and the distal convoluted tubule. A reduction in serum chloride levels stimulates renin secretion and enhances the activity of the sodium–potassium–chloride cotransporter in the thick ascending limb, as well as the thiazide-sensitive sodium–chloride symporter in the distal tubule. Consequently, low serum chloride concentrations may disrupt regulatory pathways essential for efficient renal excretion of sodium and water. Furthermore, hypochloremia is strongly associated with neurohormonal activation and diuretic resistance, which collectively impair fluid clearance in patients with HF.

Therefore, patients with diminished ALI scores and low serum chloride levels are likely to require nutritional supplementation, adjunctive pharmacotherapy, and meticulous follow-up. Electrolyte derangements and malnutrition should be considered as a crucial therapeutic focus in patients with HF. While evaluating the nutritional status of HF patients, emphasis should also be placed on the treatment modalities for those with abnormal serum chloride levels. Pharmacological intervention should be applied to recover digestive and absorptive functions, particularly in patients with gastrointestinal congestion, which may afford an opportunity to ameliorate the prognosis of HF patients and could potentially have a favorable impact on the long-term prognosis.

## 5. Limitations

Firstly, this study was a single-center, retrospective, and observational study. Hence, there may be some unmeasured variables that could potentially impact the interpretation of the research results. Secondly, this study mainly focuses on patients with a NYHA classification III or IV, moderate and severe HF. Therefore, these findings might not be applicable to a cohort with mild or moderate HF symptoms. Thirdly, we only collected data about the ALI score and serum chloride concentrations upon admission. Consequently, we were unable to examine the relationship between the dynamic changes of these two variables and the prognosis. Fourthly, notwithstanding that several covariates were taken into consideration within the regression model, it remains likely that some confounding variables are either unknown or inaccessible. Finally, we used all-cause mortality as an endpoint and did not follow up on other major cardiovascular adverse events. Prospective studies with more detailed cardiovascular endpoints could be designed in the future.

## 6. Conclusion

This study suggests that low ALI scores and low serum chloride levels are independent predictors of all-cause mortality in patients with ADHF. It is worth noting that the prognostic evaluation value of the combination of the two factors is significantly better than that of a single biomarker. Thus, the risk stratification of ADHF patients can be optimized by jointly evaluating the ALI and serum chloride levels, which helps to identify high-risk populations that require enhanced anti-inflammatory treatment and metabolic support in the early stages, ultimately improving patient prognosis.

## Availability of Data and Materials

The datasets used and analyzed during the current study are available from the corresponding author on reasonable request.
